# Histopathological evaluation of facial melasma treated with oral tranexamic acid alone and in combination with ketotifen^؆^

**DOI:** 10.1016/j.abd.2026.501336

**Published:** 2026-04-22

**Authors:** Ana Clara Ladeira Cruz, Daniel Pinho Cassiano, Hélio Amante Miot, Ana Cláudia Cavalcante Espósito, Karime Hassum, Mackerley Bleixuvehl de Brito, Milvia Maria Simões e Silva Enokihara, Maria Carolina Cavalcanti Lima Constantino, Heitor Raia Bottura, Joaquim Soares de Almeida, Ediléia Bagatin

**Affiliations:** a Department of Dermatology, Universidade Federal de São Paulo, São Paulo, SP, Brazil; b Department of Infectology, Dermatology, Imaging Diagnosis and Radiotherapy, Faculdade de Medicina, Universidade Estadual Paulista, Botucatu, SP, Brazil; c Service of Dermatology, Universidade do Oeste Paulista, Presidente Prudente, SP, Brazil; d Department of Histopathology, Universidade Federal de São Paulo, São Paulo, SP, Brazil

**Keywords:** Histology, Ketotifen, Melanosis, Tranexamic acid

## Abstract

**Background:**

Tranexamic Acid (TA) has demonstrated effectiveness on melasma treatment, and Ketotifen (KET) may inhibit mast cell-mediated melanogenesis. The histologic basis of their depigmenting effects remains unclear.

**Objectives:**

To evaluate histopathological changes from TA with KET over a 3-month treatment.

**Methods:**

In this randomized, double-blind trial, 50 women with facial melasma were assigned to KET 1 mg plus TA 250 mg (TA/KET group) or TA 250 mg (TA group), every 12 h for 3-months, with broad-spectrum sunscreen. Skin biopsies were performed at baseline and day-90. Primary outcome was epidermal melanin density reduction; secondary outcomes included stratum corneum compaction, solar elastosis, basement membrane disruptions, and counts of mast cells, melanocytes (including pendulum melanocytes), and upper dermal VEGF density.

**Results:**

Groups were comparable at baseline. Both showed reduced epidermal melanin without intergroup difference, with unchanged VEGF expression, mast cell and melanocyte count, and stratum corneum parameters. The TA/KET-group presented an increase in epidermal thickness, reduction in solar elastosis, pendulum melanocytes counting, and basal membrane disruptions.

**Study limitations:**

The short treatment and follow-up may have limited detection of histologic progression. Toluidine blue, while effective in detecting abundant mast cell populations, may lack sensitivity for precise quantification. PAS staining of the basement membrane may occasionally produce artifactual discontinuities, even when the membrane is structurally intact. Ketotifen was not tested alone.

**Conclusion:**

Both interventions led to epidermal melanin reduction. The combination TA/KET induced greater dermal and epidermal remodeling changes that surpassed those with TA alone, showing features associated with photoaging reversal and skin homeostasis restoration.

## Introduction

Melasma is a chronic, acquired pigmentary disorder characterized by symmetrical hyperpigmentation in sun-exposed areas. It predominantly affects women of childbearing age with intermediate and dark skin phototypes.^1^ In a Brazilian prevalence study, melasma was observed in 36.3% of adult women.^2^ Given its frequent facial distribution, melasma often has a significant impact on quality of life.^3^

Chronic exposure to Ultraviolet (UV) and visible light is the primary extrinsic trigger of persistent epidermal hypermelanosis in melasma. This exposure promotes the upregulation of the melanocortin-1 receptor and α-MSH, and induces a senescent phenotype in dermal fibroblasts.^4^, ^5^, ^6^ Sex hormones also contribute to the pathogenesis, as pregnancy is a commonly associated risk factor, alongside a genetic predisposition.^1^ Melanocytes in melasma are hypertrophic and contain an increased number of melanosomes. These cells undergo paracrine stimulation via cytokines and mediators such as IL-17, iNOS, endothelin, prostaglandin E2, and growth factors (e.g., α-MSH, SCF, and β-FGF), as well as by the overexpression of the estrogen receptor-β.^7^

In addition to epidermal hyperpigmentation, structural alterations resembling photoaging, such as solar elastosis, basement membrane disruption, increased vascularization, and mast cell infiltration in the upper dermis, are also observed in melasma, partly due to impaired autophagy.^8^

According to current evidence-based clinical guidelines, the first-line treatment for melasma continues to rely on the use of broad-spectrum sunscreens in combination with topical depigmenting agents.^8^, ^9^ However, the limited response in many patients has stimulated interest in adjuvant therapies, including systemic agents.

Oral Tranexamic Acid (TA), a synthetic derivative of lysine, inhibits the conversion of plasminogen to plasmin, thereby disrupting the interaction between melanocytes and keratinocytes and reducing melanin synthesis.^10^ It also suppresses the production of prostaglandins and arachidonic acid by keratinocytes, molecules known to stimulate melanogenesis. TA may further exert effects by indirectly reducing circulating α-MSH levels and acting as a competitive inhibitor of tyrosinase. Additionally, it is proposed to suppress UV-induced plasmin activity, reduce mast cell activation, and downregulate β-FGF, leading to decreased dermal vascularization and mast cell infiltration.^11^

Among the various bioactive mediators released by mast cells, histamine has been shown to promote melanogenesis primarily through H2 receptor activation.^12^ Stem Cell Factor (SCF), which is overexpressed in melasma, supports mast cell survival, proliferation, migration, and activation via binding to the c-KIT receptor. Tryptase, another mast cell-derived protease, activates Matrix Metalloproteinases (MMP-1 and MMP-9), which degrade types I and IV collagen, contributing to extracellular matrix degradation and basement membrane disruption. Mast cells also stimulate angiogenesis by releasing VEGF, FGF-2, and TGF-β.^13^, ^14^

Ketotifen Fumarate (KET) is a mast cell stabilizer commonly used in the treatment of asthma with allergic components. One proposed mechanism of action is the blockade of IgE-activated Ca²^+^ channels, which prevents the release of histamine and other mediators.^15^ In a clinical trial involving 74 women with melasma, a combination of KET and famotidine (an H2-receptor antagonist) resulted in a modest improvement in skin lightening.^16^

While the efficacy of tranexamic acid in melasma treatment is well established, the role of antihistamines remains less defined. As the histopathological basis of improvement from these agents remains poorly understood, investigating the tissue-level changes associated with KET and TA is essential for supporting the development of new treatment strategies for melasma.

## Methods

This was a prospective, randomized (1:1), comparative, parallel-group, double-blind, superiority clinical trial conducted from September to December 2022. Fifty women (aged 18–65 years) with untreated facial melasma, except for the use of sunscreen for at least one month, were enrolled. Exclusion criteria included the presence of other facial dermatoses, pregnancy or lactation, immunosuppression, and risk factors for thromboembolism (e.g., use of hormonal contraceptives or hormone replacement therapy, smoking, obesity, sedentary lifestyle, or a personal or family history of thromboembolic events).

The study was conducted at the outpatient clinic of a public hospital in Brazil. The protocol was approved by the Institutional Research Ethics Committee (approval nº 0333/2022), and all participants provided written informed consent. The study was registered in the Brazilian Registry of Clinical Trials (ReBEC: RBR-10jn7f39). Clinical, quality-of-life, and colorimetric efficacy data from this trial were published previously.^17^

Fifty eligible participants were randomized using a computer-generated sequence into two groups. The control group (TA) received 250 mg of oral tranexamic acid plus a placebo every 12 h, while the intervention group (TA/KET) received 250 mg of oral tranexamic acid plus 1 mg of ketotifen every 12 h. All components were compounded into identical capsules, and both participants and evaluators were blinded to treatment allocation. The blinding code was maintained by the compounding pharmacy and was only broken after statistical analyses were completed. Systemic treatment lasted 90-days.^18^ All participants received a tinted broad-spectrum sunscreen (Photoaging Control SPF 50, Eucerin™) and were instructed to reapply it every 3-hs during the day throughout the study period.

Skin biopsies were obtained using a 3-mm punch at baseline (D0) and on day-90 (D90), from the same anatomical site, with a maximum variation of 1 cm. Samples were fixed in formalin, paraffin-embedded, and stained with Hematoxylin-Eosin (HE), Fontana-Masson, Periodic Acid-Schiff (PAS), and toluidine blue. Immunohistochemistry was performed using anti-Melan-A (Dako, undiluted, A103 clone, RTU) and anti-vascular endothelial growth factor (VEGF; Dako, 1:50 dilution, VG1 clone) antibodies.

Slides were photographed in triplicate at 40× magnification using a digital scanner (3DHistech, Budapest, Hungary), selecting representative interfollicular areas free of artifacts. Images were saved in TIFF format. Quantitative analyses were conducted using ImageJ software (version 1.51e; NIH, USA) by a trained dermatologist who was blinded to both the biopsy time point (D0 or D90) and the treatment allocation. The only exception was the Toluidine Blue staining, which was evaluated by two trained pathologists due to the complexity of its assessment.^19^

The primary outcome was the change in epidermal melanin content between D0 and D90, assessed by Fontana-Masson staining using split-channel analysis and image binarization. Staining intensity was quantified as the mean pixel intensity in the color histogram, ranging from 0 (black) to 255 (white). Secondary outcomes included: stratum corneum compaction and solar elastosis (evaluated on HE); basement membrane disruptions (PAS); mast cell density in the upper dermis (toluidine blue); melanocyte density and presence of pendulum melanocytes (Melan-A); and VEGF staining intensity (color deconvolution analysis in ImageJ). Due to the algorithm used for deconvolution in ImageJ, intensity values are not restricted to the standard 8-bit scale (0–255), but reflect summed optical density, yielding higher raw pixel values proportional to stain deposition.^20^ Solar elastosis was qualitatively graded as: 0 = absent, 1 = mild/focal to moderate, and 2 = intense. Stratum corneum compaction was scored as: 0 = absent, 1 = partial, and 2 = diffuse.^21^

Sample size was calculated using G*Power software to detect a between-group difference greater than 20%, assuming a standard deviation of a similar magnitude. A 10% dropout rate was anticipated. With a one-tailed alpha of 0.05 and 80% statistical power, the required sample was 25 participants per group.

Normality was assessed using the Shapiro-Wilk test.^22^ Statistical analyses were performed using IBM SPSS version 25. Between-group comparisons were conducted using a generalized linear mixed-effects model with Sidak adjustment for multiple comparisons.^23^ Analyses followed the intention-to-treat principle, and missing data were handled via mixed model imputation. Statistical significance was set at p ≤ 0.05 (one-tailed).^24^

## Results

**Fig. 1:**
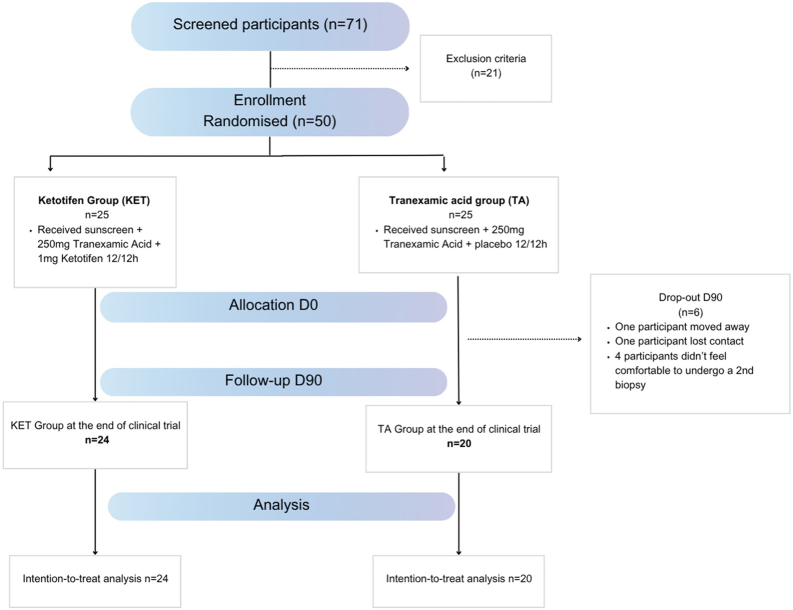
CONSORT flow diagram of the study.

A total of 71 participants were initially screened, of whom 21 (29.5%) were excluded based on eligibility criteria. The trial ultimately enrolled 50 participants, equally allocated to the TA group (n = 25) and the TA/KET group (n = 25). Six participants discontinued the study (5 from the TA group and 1 from the TA/KET group) due to reasons unrelated to treatment-related adverse effects: two were lost to follow-up, and four declined to undergo the second biopsy (Fig. 1).

**Fig. 2:**
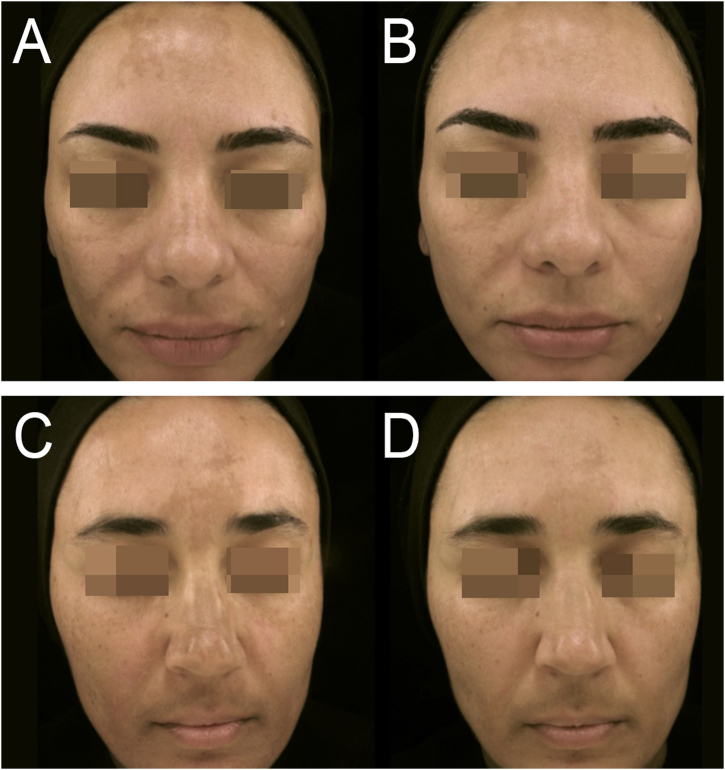
Photographic records are presented at baseline (DO), at the conclusion of systemic treatment (D90). TA group (A‒B); KET group (C‒D).

The groups were demographically comparable: the mean age (SD) was 48 (6) years in the TA/KET group and 45 (7) years in the TA group, and most participants in both groups had high phototypes (IV, V, or VI) (Table 1). Both groups showed clinical improvement in all evaluated parameters (mMASI, MelasQoL, and colorimetry) from baseline (D0) to post-treatment (D90), with no differences between them (Fig. 2). At day 90 (D90), for example, the mean reduction in mMASI was 47% (36–58%) in the TA group and 52% (44%–59%) in the KETO group (p > 0.25, 95% CI). During follow-up without treatment (D120), recurrence was observed in both groups, with no significant difference between them (p = 0.36).^17^

**Table 1:** Main clinical and demographic variables of the participants at inclusion.

Variables		TA/KET group	TA group
n		25	25
Age (years), mean (SD)		48 (6)	45 (7)
Phototype, n (%)	II and III	4 (16)	9 (36)
	IV	11 (44)	9 (36)
	V and VI	10 (40)	7 (28)
Pregnancies, median (P_25_‒P_75_)		2 (1–2)	2 (1–3)
Daily exposition to the sun (min), mean (SD)		95 (79)	73 (68)
Family history of melasma, n (%)		18 (72)	17 (68)
Age of melasma onset (years), mean (SD)		33 (11)	29 (9)
mMASI, mean (SD)		8 (2)	8 (2)
MELASQoL, mean (SD)		47 (12)	53 (12)
Dif-*L, mean (SD)		4 (2)	4 (2)

Group TA/KET: Combination therapy of oral 1 mg of ketotifen plus 250 mg of tranexamic acid 12/12 h. Group TA: Oral therapy of 250 mg of tranexamic acid plus placebo 12/12 h. mMASI, Modified Melasma Area and Severity Index; MELASQoL, Melasma Quality of Life questionnaire; Dif-*L, Colorimetric difference of the lightness (*L) between melasma and adjacent skin.

**Fig. 3:**
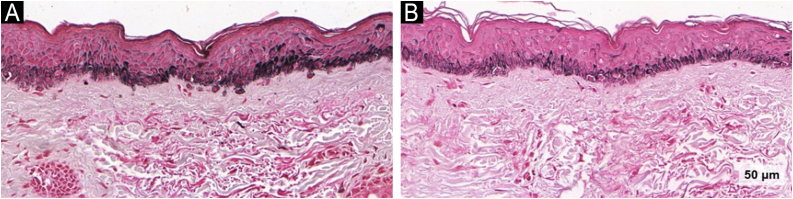
Fontana-Masson images of TA group evidencing epidermal melanin reduction: (A) baseline and (B) post-treatment (Fontana-Masson, ×40).

Histological and immunohistochemical findings are summarized in Table 2. An overall reduction in epidermal melanin content was observed in both groups after 90-days of treatment (−12.7; 95% CI: −18.0 to −7.4; p < 0.001), with no difference between the groups (Fig. 3).

**Table 2:** Histological and immunohistochemical variables according to group (n = 44).

Variables	TA			TA/KET			p (time × group)
	D0	D90	p (time)	D0	D90	p (time)	
	Mean (SD)			Mean (SD)			
Epidermal melanin (intensity)^a^	84 (13)	67 (16)	<0.001	83 (13)	75 (17)	0.005	0.103
Dermal melanin (intensity)^a^	21 (14)	13 (7)	0.013	19 (14)	18 (12)	0.74	0.183
Mast cells^b^	1.7 (1.9)	2.8 (2.9)	0.154	2.7 (2.2)	2.2 (1.5)	0.253	0.424
Basal layer melanocytes^c^	16 (5)	14 (5)	0.131	17 (6)	15 (4)	0.085	0.480
Pendulum melanocytes^c^	1 (0.8)	0.8 (1.3)	0.219	1.5 (1.3)	1 (1.2)	0.015	0.54
Melanocytes area (×10)^d^	68 (19)	63 (12)	0.344	71 (24)	57 (14)	0.034	0.179
BMZ discontinuities^c^	0.9 (0.8)	0.8 (0.8)	0.776	1.3 (0.9)	0.6 (0.6)	<0.001	0.608
Thickness of the stratum corneum [μm]	40 (12)	43 (16)	0.18	44 (16)	47(13)	0.5	0.335
Thickness of the epidermis [μm]	97 (26)	261 (35)	0.156	282 (30)	264 (33)	<0.001	0.191
Compaction of the stratum corneum^e^	**n (%)**			**n (%)**			
0 (absent)	10 (40)	10 (47)	0.387	12 (48)	16 (66)	0.257	0.323
1 (partial)	11 (44)	9 (42)		11 (44)	6 (25)		
2 (diffuse)	4 (16)	2 (9)		2 (8)	2 (8)		
Solar elastosis^f^	**n (%)**			**n (%)**			
0 (absent)	3 (12)	4 (19)	0.166	4 (16)	5 (20)	0.014	0.636
1 (mild to moderate)	13 (52)	11 (52)		12 (48)	14 (58)		
2 (moderate to severe)	9 (36)	6 (28)		9 (36)	5 (20)		
VEGF ×103 intensity^g^	79 (69)	77 (13)	0.465	73 (68)	76(11)	0.276	0.683

a Mean pixel intensity, as shown in the color histogram, varies between 0 and 255.

b Number of mast cells (calculated as the mean count across five zones at 40× magnification).

c Absolute number of cells at 40× magnification.

d Melanocytes mean area at 40× magnification.

e Visual qualitative scale: 0 (compactation absent, “basket-weave” pattern), 1 (partial corneum compaction, intermixed areas of compact and non-compact stratum corneum) and 2 (diffuse compaction of the stratum corneum).

f Visual qualitative scale for solar elastosis in the upper dermis: 0 (absent), 1 (mild to moderate) and 2 (moderate to intense).

g Vascular Endothelial Growth Factor (VEGF, Dako, 1:50): mean pixel intensity in the upper dermis. Due to the output scale of the deconvolution algorithm, values were no longer restricted to the standard 8-bit range (0–255) and instead ranged between 73,000 and 7.

**Fig. 4:**
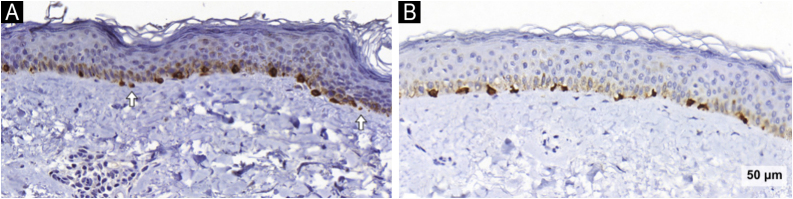
Melan-A images of the TA/KET group. Arrows indicate pendulous melanocytes before (A) and reduced after treatment (B) (Melan-A, ×40).

The melanocyte count, assessed via Melan-A staining, remained unchanged in both groups. However, a reduction in melanocyte area was observed across groups after treatment (−89; 95% CI: −167 to −11; p = 0.025), again with no between-group difference. Notably, melanocyte area decreased significantly in the TA/KET group (−132; 95% CI: −254 to −10; p = 0.034). In addition, the number of pendulum melanocytes was reduced in the TA/KET group (−0.66; 95% CI: −1.18 to −0.13; p = 0.015) (Fig. 4).

**Fig. 5:**
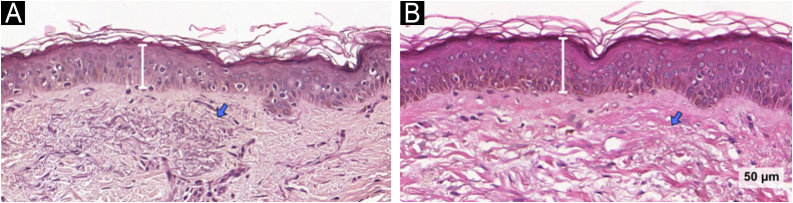
Hematoxylin & eosin images of the TA/KET group: (A) before, (B) after. Bars indicate the increased epidermal thickness; arrows show reduced solar elastosis (Hematoxylin & eosin, ×40).

**Fig. 6:**
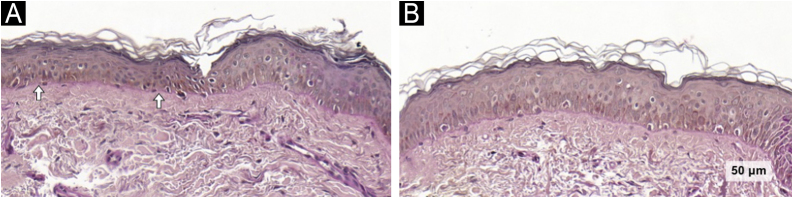
PAS images of the TA/KET group: (A) before, (B) after. Arrows highlight areas of basement membrane discontinuity restored after treatment (40× magnification).

An increase in epidermal thickness was observed exclusively in the TA/KET group at day-90 (14.3; 95% CI: 7.1 to 21.4; p < 0.001). This group also showed a significant reduction in solar elastosis (-0.20; 95% CI: −0.41 to −0.36; p = 0.014), suggesting a favorable remodeling effect in the dermal compartment (Fig. 5). Furthermore, the number of basal membrane disruptions decreased significantly only in the TA/KET group after treatment (−0.73; 95% CI: −1.04 to −0.4; p < 0.001) (Fig. 6).

No significant changes were observed between groups or over time in mast cell count (evaluated using toluidine blue staining), stratum corneum thickness and compaction (assessed via H&E), or VEGF expression (evaluated by immunostaining).

## Discussion

Although no clinical differences were observed between groups after treatment, and a relapse occurred in both groups 30-days after treatment suspension,^17^ this study revealed distinct histopathological changes following the interventions, suggesting a potential role of mast cell stabilizers and antihistamines in the treatment of melasma.

The pathogenesis of melasma involves multiple mechanisms beyond melanocyte hypertrophy. Alterations in different skin compartments, such as the epidermis, dermis, basement membrane, and cutaneous barrier, are involved in the disease process.^7^ Histological features such as solar elastosis, increased perivascular mast cells and sebaceous glands, basement membrane disruption, and enhanced vascularization have been consistently demonstrated in melasma-affected skin.^25^, ^26^, ^27^, ^28^ Additionally, a transcriptional analysis of melasma lesions reported the involvement of nearly 300 genes, underscoring the complexity of its pathophysiology, which extends beyond melanocytes to include dermal components as well.^29^

The reduction in epidermal melanin observed in both the TA and TA/KET groups is consistent with previous evidence supporting the depigmenting effects of TA, even when used without topical lightening agents.^10^, ^11^ However, the addition of ketotifen did not yield a significantly greater reduction in melanin, possibly due to the dominant effect of TA masking any additional impact. Notably, although between-group differences in melanocyte volume were not statistically significant, the TA/KET group showed a more pronounced reduction after treatment.

While melanophages are commonly increased in hyperpigmented skin,^30^, ^31^ their exact role in melasma remains uncertain. Given their absolutely low density compared to epidermal melanin,^32^ their contribution may be more indicative of chronic photodamage than a primary driver of pigmentation. Nevertheless, melanophages are recognized markers of photoaging and are more prevalent in individuals with darker phototypes.^30^, ^31^

Although total melanocyte counts remained stable in both groups, a significant reduction in pendulum melanocytes was observed in the TA/KET group. These cells are more prevalent in melasma lesions compared to perilesional skin^33^ and are supposed to represent inactive dendritic melanocytes, as demonstrated by confocal microscopy.^26^ Lacking epidermal connectivity, they are unlikely to contribute directly to melanogenesis but can be induced by UVA radiation.^34^ While the precise mechanisms underlying their formation are unclear, evidence suggests that chronic sun exposure may increase MMP-2 expression, promoting basement membrane disruption and pendulum melanocyte formation.^35^

Mast cell density is typically higher in chronically sun-exposed areas compared to non-exposed regions,^36^ and they induce melanogenesis via bioactive mediators acting on histamine H2 receptors.^12^ The role of mast cells in photoaging is further supported by experimental studies in UV-irradiated mice, in which ketotifen treatment reduced wrinkle formation, epidermal thinning, mast cell accumulation, and matrix degradation.^15^ Although mast cell counts did not change in the present study, several histological markers of photoaging improved only in the TA/KET group, including reduced solar elastosis, restoration of basement membrane integrity, decreased pendulum melanocyte presence, and increased epidermal thickness.

The maintenance of dermal and epidermal homeostasis is critically dependent on the integrity of the basement membrane. In melasma, this structure is often disrupted, facilitating the diffusion of dermal cytokines into the epidermis, which enhances melanogenesis and supports melanocyte protrusion into the dermis. In addition, fibroblasts in the melasma dermis exhibit a senescent phenotype with impaired capacity for basement membrane repair.^37^ In the present study, treatment with TA/KET led to a significant reduction in basement membrane disruptions, an effect not observed in the TA group.

In a cross-sectional study of 50 patients with facial melasma, epidermal atrophy was observed in 90% of lesions versus 28% of perilesional skin samples.^32^ Similarly, the authors observed increased epidermal thickness after treatment in the TA/KET group. A recent study proposed that a thicker epidermis may enable a more uniform distribution of melanin, improving the absorption of solar radiation by photochromophores and reducing direct UV impact on melanocytes, thereby attenuating melanogenesis.^6^

Solar elastosis, a hallmark of chronic UV exposure, is more prevalent in melasma-affected skin compared to adjacent areas, reinforcing the hypothesis that photoaging contributes to melasma pathogenesis. UVB exposure stimulates keratinocytes to promote melanocyte proliferation via secretion of SCF, βFGF, and α-MSH.^5^ In this trial, the reduction in solar elastosis observed in the TA/KET group suggests that ketotifen may exert a beneficial remodeling effect on the dermal matrix.

Melasma is associated with increased vascularity, which is not merely a consequence of UV damage but appears to play a central role in disease pathogenesis. VEGF, known to influence melanocyte activity through its receptor, is upregulated in melasma lesions.^38^ In this study, however, VEGF expression did not decrease in either group, indicating that TA is ineffective to act in this aspect.

The stratum corneum in melasma is typically more compact, and improving its integrity can enhance protection against UV radiation. Treatments such as triple combination creams and procedures like chemical peels or lasers have demonstrated efficacy in reducing stratum corneum compaction.^4^, ^34^ In the present study, no significant changes in the stratum corneum were observed in either group, potentially due to the absence of adjunctive topical therapies or resurfacing interventions.

Finally, as melasma is a multifactorial condition involving multiple skin compartments and pathogenic pathways, the most effective interventions should target multiple fronts to restore skin homeostasis and promote long-lasting results. Mast cells can stimulate melanogenesis through the release of bioactive mediators acting on histamine H2 receptors. Ketotifen, a mast cell stabilizer, has demonstrated potential skin-rejuvenating effects, including increased epidermal thickness and reductions in solar elastosis, pendulum melanocytes, and basement membrane disruption. These results are consistent with previous studies showing ketotifen’s ability to prevent UV-induced wrinkle formation in murine models and further support its potential to improve photoaging-related parameters in melasma-affected skin.^15^

Since the combination of oral TA/KET was not superior to oral TA alone in terms of clinimetric parameters,^17^ this association should be tested in clinical trials with different regimens before it can be recommended in clinical practice. Notably, the topical application of ketotifen remains largely unexplored, highlighting the need for future studies integrating it into combination treatment strategies.

### Limitations

The use of 3 mm biopsies may not represent the whole melasma across the face. The relatively short duration of the trial may also have limited the observation of robust dermal remodulation despite the histopathological changes found. Ketotifen was not assessed without its combination with TA. Toluidine blue, while effective in detecting abundant mast cell populations, may compromise the sensitivity for precise quantification of sparse cells. PAS staining of the basement membrane may occasionally produce artifactual discontinuities, even when the membrane is structurally intact; however, it did not hinder the detection of changes.

## Conclusion

In this trial, both interventions led to a reduction of epidermal melanin. The combination of KET with TA produced histopathological changes indicative of dermal and epidermal remodeling that surpassed those observed with TA alone, including increased epidermal thickness, reduced solar elastosis, fewer pendulum melanocytes, and improved basement membrane integrity, features associated with photoaging reversal and restoration of skin homeostasis. These findings suggest that targeting mast cell-mediated pathways may offer structural benefits beyond pigment reduction, potentially addressing deeper pathogenic components of melasma.

## ORCID ID

Daniel Pinho Cassiano: 0000-0003-2615-0456

Hélio Amante Miot: 0000-0002-2596-9294

Ana Cláudia Cavalcante Espósito: 0000-0001-9283-2354

Karime Hassum: 0000-0003-1143-9521

Mackerley Bleixuvehl de Brito: 0009-0008-5843-9143

Milvia Maria Simões e Silva Enokihara: 0000-0002-3340-4074

Maria Carolina Cavalcanti Lima Constantino: 0009-0001-6006-3041

Heitor Raia Bottura: 0000-0002-7723-5807

Joaquim Soares de Almeida: 0009-0005-8437-1726

Ediléia Bagatin: 0000-0001-7190-8241

## Registration

This trial was registered on Plataforma Brazil (https://plataformabrasil.saude.gov.br) under the number CAAE 57773122.9.0000.5505.

This protocol was registered by the ReBEC platform (Brazilian registry of Clinical Trials) under the number ReBEC: RBR-10jn7f39.

## Financial support

The trial had major funding from FUNADERSP (Dermatology support fund of the state of São Paulo) under the number 109/22.

The donation of sunscreens was made by the company Eucerin and of tranexamic acid by Galena Farmacêutica.

## Authors' contributions

Ana Clara Ladeira Cruz: Participant recruitment and clinical assessment; analysis and interpretation of clinical and histopathological data; funding acquisition; manuscript drafting and critical revision; final approval of the manuscript.

Daniel Pinho Cassiano: Co-supervision; study conception and design; analysis and interpretation of clinical and histopathological data; funding acquisition; critical revision of the manuscript; final approval of the manuscript.

Hélio Amante Miot: Co-supervision; statistical analysis; critical revision of the manuscript; final approval of the manuscript.

Ana Cláudia Cavalcante Espósito: Critical revision of the manuscript; final approval of the manuscript.

Karime Hassun: Funding acquisition; Critical revision of the manuscript; final approval of the manuscript.

Mackerley Bleixuvehl de Brito: Clinical assessment of participants; final approval of the manuscript.

Milvia Maria Simões e Silva Enokihara: Histopathological analysis; final approval of the manuscript.

Maria Carolina Cavalcanti Lima Constantino: Histopathological processing; final approval of the manuscript.

Heitor Raia Bottura: Histopathological analysis; final approval of the manuscript.

Joaquim Soares de Almeida: Histopathological processing; final approval of the manuscript.

Ediléia Bagatin: Supervision; Critical revision of the manuscript; final approval of the manuscript.

## Research data availability

The entire dataset supporting the results of this study was published in this article.

## Conflicts of interest

None declared.
